# Myocardial lipofuscin accumulation in ageing and sudden cardiac death

**DOI:** 10.1038/s41598-019-40250-0

**Published:** 2019-03-01

**Authors:** Yu Kakimoto, Chisa Okada, Noboru Kawabe, Ayumi Sasaki, Hideo Tsukamoto, Ryoko Nagao, Motoki Osawa

**Affiliations:** 1 Department of Forensic Medicine, Tokai University School of Medicine, Kanagawa, Japan; 2 Support Center for Medical Research and Education, Tokai University, Kanagawa, Japan

## Abstract

Lipofuscin is an intracellular aggregate of highly oxidized proteins that cannot be digested in the ubiquitin-proteasome system and accumulate mainly in lysosomes, especially in aged cells and pathological conditions. However, no systematic study has evaluated the cardiac accumulation of lipofuscin during human ageing and sudden cardiac death (SCD). Age estimation in unidentified bodies and postmortem SCD diagnosis are important themes in forensics. Thus, we aimed to elucidate their correlations with myocardial lipofuscin accumulation. We collected 76 cardiac samples from autopsy patients aged 20–97 years. After histopathological examination, myocardial lipofuscin was measured using its autofluorescence. Lipofuscin accumulated mainly in the perinuclear zone, and its accumulation rate positively correlated with chronological ageing (r = 0.82). Meanwhile, no significant change in lipofuscin level was observed with different causes of death, including SCD. There was also no significant change in lipofuscin level in relation to body mass index, serum brain natriuretic peptide level, or heart weight. Moreover, we performed LC3 and p62 immunoblotting to evaluate autophagic activity, and no change was observed in ageing. Therefore, lipofuscin accumulation more directly reflects chronological ageing rather than human cardiac pathology. Our study reveals the stability and utility of cardiac lipofuscin measurement for age estimation during autopsy.

## Introduction

Lipofuscin is a yellow-brown pigment composed of highly oxidized proteins, lipids, and metals^1–3^. Lipofuscin accumulation is enhanced under oxidative stress^4,5^, and reactive oxygen species produced by damaged mitochondria also contribute to lipofuscin formation^6,7^. Because lipofuscin is a covalently cross-linked aggregate, it cannot be removed from the cytosol by the ubiquitin-proteasome system^8,9^. Cytosolic lipofuscin is taken up by autophagosomes and eventually accumulates in lysosomes^10^. Lipofuscin is widely observed in postmitotic cells, especially in long-lived cells such as neurons and cardiomyocytes. However, the composition of lipofuscin is heterogeneous among tissue types and biological species^11,12^. Because there is no specific antibody for lipofuscin, using lipofuscin autofluorescence is the standard method of detection and quantification^4,7,10,13,14^. Other classical histochemical techniques are also applicable for lipofuscin observation, including haematoxylin and eosin, Sudan black, Fontana-Masson, and Schmorl stains^15–17^. Although lipofuscin has been known as an age pigment, which accumulates in ageing, no systematic analysis of lipofuscin accumulation in human cardiac ageing has been reported.

To date, the pathogenic roles of lipofuscin have been strongly suggested in various diseases. Lipofuscin can be cytotoxic partly because of its ability to incorporate transition metals such as iron and copper, resulting in a redox-active surface^2,18^. Intracellular lipofuscin interferes with the ubiquitin-proteasome system and autophagy-lysosomal pathway^8,14,19^. These clearance systems are essential for the removal of damaged organelles and oxidized proteins, and impairment in these systems could exacerbate lipofuscin accumulation and reduce cellular viability^10,20^. Prominent accumulation of lipofuscin has been observed in many neurodegenerative diseases, including Alzheimer’s disease and Parkinson’s disease^21–24^. The elevation of lipofuscin accumulation levels has been also reported in the end stage of heart failure with dilated cardiomyopathy and ischemic cardiomyopathy^25–27^. Thus, lipofuscin accumulation is considered a hallmark of chronic degenerative diseases, while the lipofuscin accumulation level in sudden cardiac death (SCD) without preceding heart failure symptoms has yet to be elucidated.

Here, we focused on myocardial lipofuscin accumulation from the forensic aspect. First, we evaluated the correlation between myocardial lipofuscin levels and ageing in normal human hearts. Estimation of chronological age is important at autopsy of unidentified remains and in mass disasters. Age prediction trials have been performed using pulp/tooth volume, telomere length, and DNA methylation patterns^28–30^. These approaches require imaging such as computed tomography and magnetic resonance imaging or additional DNA analyses, while lipofuscin assay can be performed in parallel with routine histochemical examinations. Subsequently, we have analysed the myocardial lipofuscin accumulations in various causes of death including SCD, the major cause of sudden internal death. It is often difficult to diagnose SCD without macroscopical changes at autopsy, because contributing lethal arrhythmia is not directly detectable at postmortem^31–33^. Lipofuscin is an nondegradable final aggregate that is stably detected even at autopsy. Therefore, lipofuscin quantification can be worthy of trial in forensics.

## Methods

### Subjects

Cardiac tissues were collected from 76 subjects at forensic autopsy (Table 1, Supplemental Table 1). The average age was 59.1 years (range, 20–97 years). Subjects were divided into seven groups according to the cause of death: accident (Acc), ischemic heart failure (IHF), hypertrophic heart failure (HHF), cancer (Ca), brain haemorrhage (Br), hepatic failure (Hep), and other diseases (Dis). Acc cases were those who died in accidents without severe cardiac pathology. IHF and HHF cases were SCD cases who could perform normal daily activities at least 24 hours prior to death. IHF cases showed lethal coronary artery occlusions, while HHF cases showed cardiac hypertrophy without severe coronary atherosclerosis. Cardiac hypertrophy was defined as heart weight exceeding 400 g, which approximately corresponds to heart weight/body height of >2.5 g/cm in Japanese people^34^. The HHF group consisted of six hypertensive heart failure cases and two aortic stenosis cases, whereas the Ca group consisted of four gastric cancer cases and a breast cancer case. The Br group comprised two pontine haemorrhages, two putamen haemorrhages, and a thalamic haemorrhage. All Hep cases experienced alcohol cirrhosis without cancers. The Dis group consisted of three sepsis cases, three ileus cases, two pneumonia cases, and an enterogastritis case. There were no significant differences in age, body mass index (BMI), and serum brain natriuretic peptide (BNP) level among the groups. Heart weight was significantly increased in the HHF group.

**Table 1:** Clinical and histological characteristics of subjects.

	Acc	SCD		Non-cardiac disease			
		IHF	HHF	Ca	Br	Hep	Dis
No. of cases	35	10	8	5	5	4	9
Gender (M/F)	25/10	6/4	8/0	3/2	4/1	4/0	5/4
Age (years)	58.6 ± 18.8	64.2 ± 18.7	65.8 ± 18.3	64.4 ± 19.5	59.0 ± 19.2	51.5 ± 18.0	60.7 ± 18.0
BMI (kg/m^2^)	22.0 ± 3.7	22.1 ± 3.5	30.7 ± 5.7	16.3 ± 2.5	19.6 ± 3.0	20.8 ± 2.5	20.9 ± 3.9
BNP (pg/dl)	17.5 ± 34.0	80.4 ± 94.9	125 ± 165	—	—	—	50.5 ± 47.5
Heart weight (g)	384 ± 96.2	409 ± 96.9	630 ± 137*	318 ± 87.2	414 ± 84.1	332 ± 84.1	371 ± 99.2

Acc, accidents; SCD, sudden cardiac death; IHF, ischemic heart failure; HHF, hypertrophic heart failure; Ca, cancer; Br, brain haemorrhage; Hep, hepatic failure; Dis, other diseases; M/F, male/female; BMI, body mass index; BNP, brain natriuretic peptide. *P < 0.01.

This study was approved by the Ethics Committee of Tokai University, and the study protocol conformed to the ethical guidelines of the 1975 Declaration of Helsinki. Written informed consent allowing the experimental use of the tissue samples was obtained from the bereaved relatives of all subjects.

### Tissue preparation

Cardiac transverse sections were obtained at middle height between the aortic root and apex. The cardiac tissues were fixed with 10% formalin and embedded in paraffin. Then, 4-μm thick sections from the whole area of the both ventricles were stained with haematoxylin and eosin dyes for histopathological examination, and 2.5-μm-thick sections from the free wall of the left ventricle were used for lipofuscin assay without staining. Other small samples of the free wall of the left ventricle were frozen and stored at −80° prior to immunoblotting.

### Lipofuscin assay

The middle layer (myocardium) of the heart was used for lipofuscin quantification, wherein cardiomyocytes showed uniform alignment of longitudinal sections. Severely damaged areas with trauma or ischemia were avoided because measurements of these areas may be influenced by morphological changes. Autofluorescence of myocardial lipofuscin was detected using an Axio Imager M2 microscope with Axiocam 506 mono camera (Carl Zeiss Microscopy GmbH, Jena, Germany). Lipofuscin exhibits broad-spectrum autofluorescence, with peak emission wavelengths of 550 and 650 nm^35^; a DsRed filter set was applied with an exposure time of 500 ms throughout the observation. The areas of lipofuscin accumulation and the myocardia were measured using Fiji/ImageJ 1.52e (National Institutes of Health, Bethesda, MD, USA). Ten fields per subject were observed at 20× objective magnification, and the lipofuscin accumulation rate (lipofuscin deposit area/myocardial area) was calculated.

### Immunoblotting

Approximately 150 mg of cardiac tissue was homogenised in RIPA buffer and centrifuged to yield supernatant. Thirty micrograms of supernatant proteins were subjected to 15% acrylamide gel electrophoresis and transferred to polyvinylidene difluoride membranes. The membranes were then incubated overnight at 4 °C with primary antibodies against LC3 (Sigma; L8918; 1:1000) and p62 (Progen; 703251; 1:1000). GAPDH (Sigma; G9545; 1:3000) was used as an internal loading control. The blots were visualized using a detection kit (Immobilon).

### Statistics

All data were presented as means ± standard deviations. Multiple comparisons of the clinical characteristics and lipofuscin levels in each group were performed using Steel’s test. The Acc group was used as a control. The correlation between age and lipofuscin accumulation was assessed using a correlation coefficient (r). The proportion of total variation was explained by an age prediction model and assessed by the coefficient of determination (R^2^). Multiple comparisons about absolute error of predicted ages were performed with Steel’s test using the <40-year group as control. After excluding Ca cases, the other cases were divided into two age-matched groups according to BMI and BNP levels, and heart weight and the lipofuscin levels were compared using Welch’s t-test. Differences with P values of <0.01 were considered statistically significant throughout this study.

## Results

### Histopathological findings

**Figure 1:**
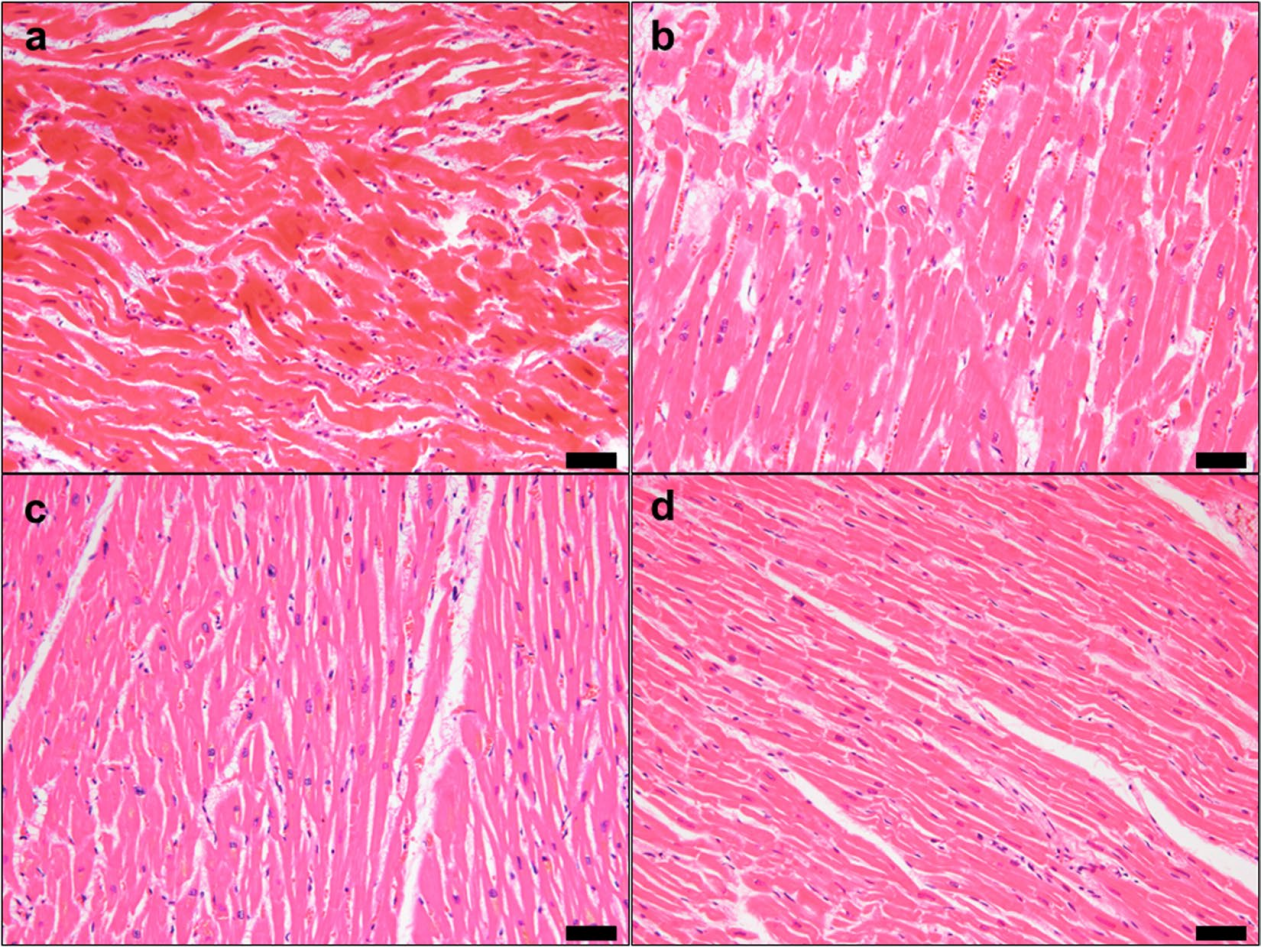
Histopathological findings of cardiac samples. Representative findings with haematoxylin and eosin staining. (**a**) Ischemic heart failure case (45-year-old female). Coagulation necrosis is ongoing. Hypereosinophilic myocardia show wavy changes with early neutrophilic infiltrate. (**b**) Hypertrophic heart failure case (46-year-old male). Hypertrophic myocytes with enlarged nuclei are regularly arrayed with slight interstitial fibrosis. (**c**) Elderly accident case (88-year-old female). A moderate level of interstitial fibrosis is shown. (**d**) Young accident case (23-year-old male). Myocardial structure is maintained without pathology. Scale bar indicates 50 μm.

Coagulation necrosis, contraction band necrosis, wavy fibres, myocyte hypereosinophilia, and scattered neutrophils were observed in IHF (Fig. 1a). Cardiomyocytes and nuclei were equally enlarged in HHF and Br (Fig. 1b), but asymmetric septal hypertrophy or myofibre disarray, which is characteristic of hypertrophic cardiomyopathy, was not observed in all cases. Low to moderate levels of interstitial fibrosis were observed in some IHF, HHF, Br, and elderly subjects (Fig. 1c). Other cases preserved the normal myocardial structures (Fig. 1d). There was no massive inflammation in all cases.

### Lipofuscin accumulation assays

**Figure 2:**
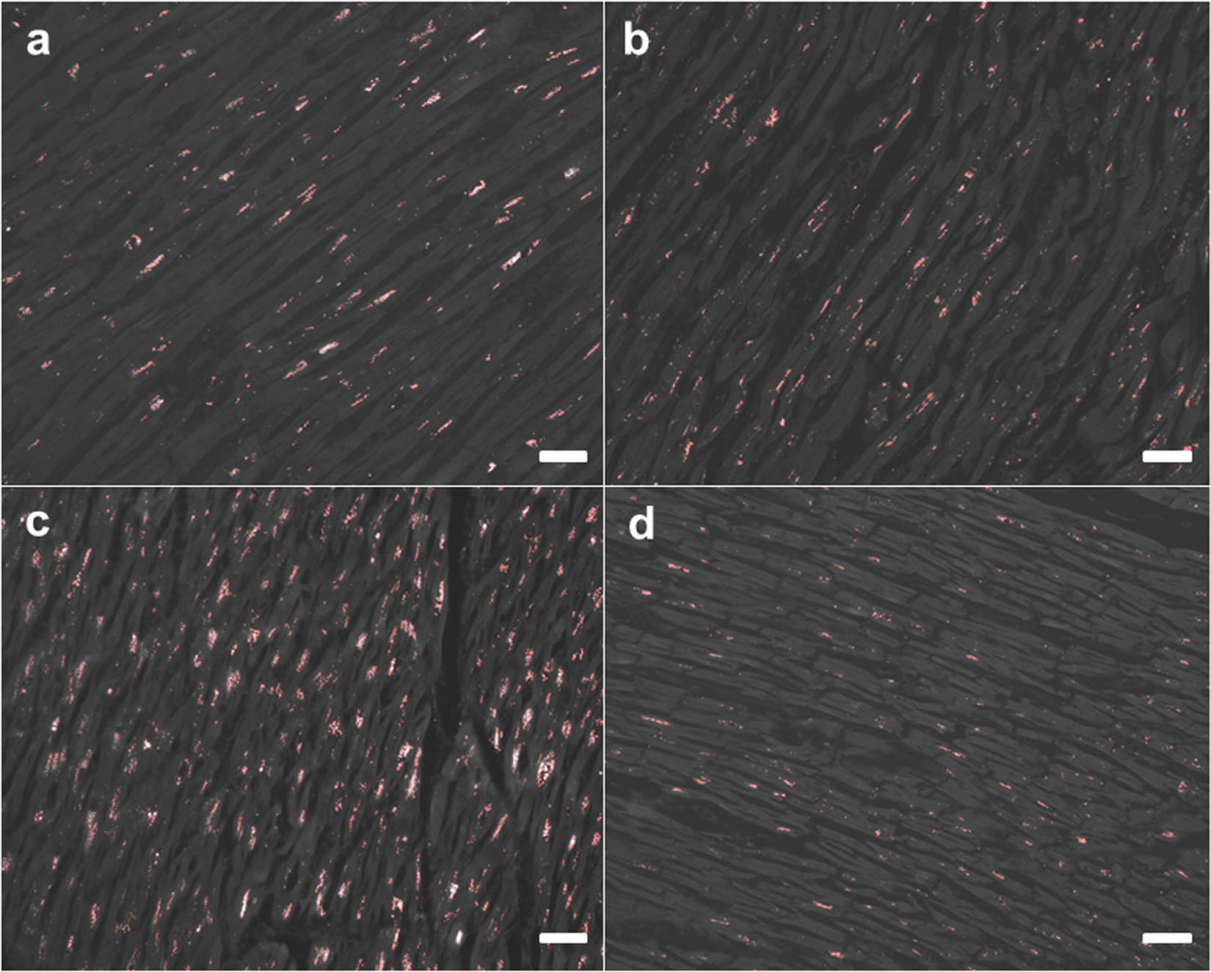
Lipofuscin accumulation assays. Identical specimens as in Fig. 1. observed by fluorescence microscope. (**a**,**b**) Samples of middle-aged cases showing moderate levels of lipofuscin deposit, independent of cardiac pathology. (**c**) Sample of an aged case showing excessive lipofuscin accumulation. (**d**) Sample of a young case showing small perinuclear granules of lipofuscin. Scale bar indicates 50 μm.

**Figure 3:**
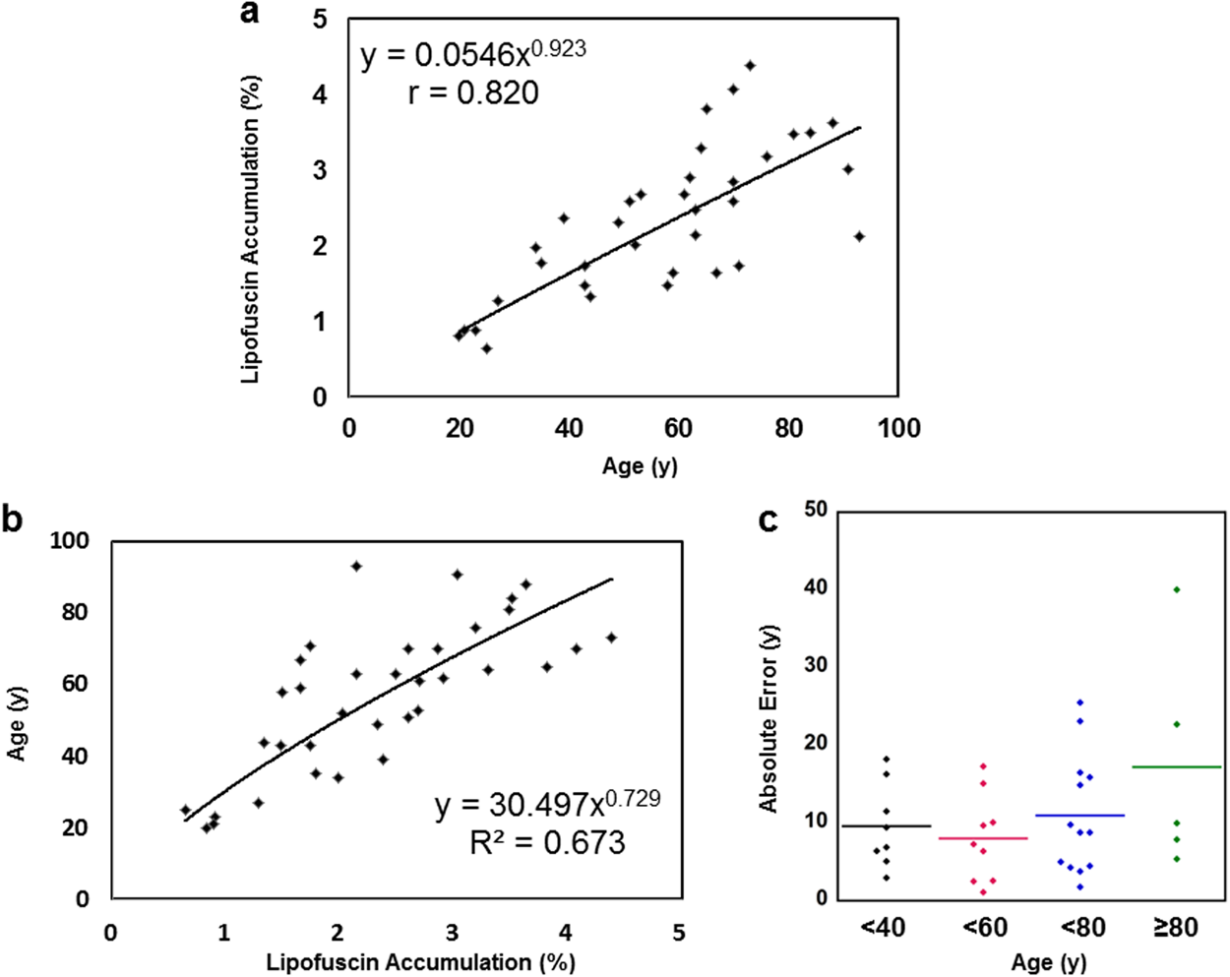
Cardiac lipofuscin accumulation and chronological ageing. Normal human hearts obtained from accidental death cases were analysed (n = 35). (**a**) Lipofuscin accumulated in myocardia with a strong correlation with ageing. (**b**) Chronological ages can be predicted using myocardial lipofuscin accumulation levels. (**c**) The mean absolute error of age prediction using myocardial lipofuscin level is presented according to <40−, <60−, <80−, and ≥80-year groups (n = 8, 9, 13, and 5, respectively). Horizontal bars indicate the mean values.

Lipofuscin accumulated mainly in the perinuclear area in the centre of myocytes and scattered in the cytosol as small granules (Fig. 2a–d). The lipofuscin accumulation rate ranged from 0.6% to 7.0% among subjects, and the standard deviation of lipofuscin accumulation per field in each subject was 0.6% (Supplemental Table 2). The deposit pattern did not apparently change according to ageing or cardiac pathology, while the total amount of lipofuscin increased according to ageing in Acc subjects (Fig. 3a). Regression analysis revealed that the chronological age could be estimated using the myocardial lipofuscin level as follows (Fig. 3b):

y = 30.497x^0.729^, where y is age (years), x is lipofuscin accumulation (%), and R^2^ = 0.673.

The mean absolute error was 10.8 years across all age groups and 9.6, 8.0, 11.0, and 17.2 years in the <40−, <60−, <80−, and ≥80-year groups, respectively (Fig. 3c). Although there was no significant change among the age groups, the error was larger in older cases.

**Figure 4:**
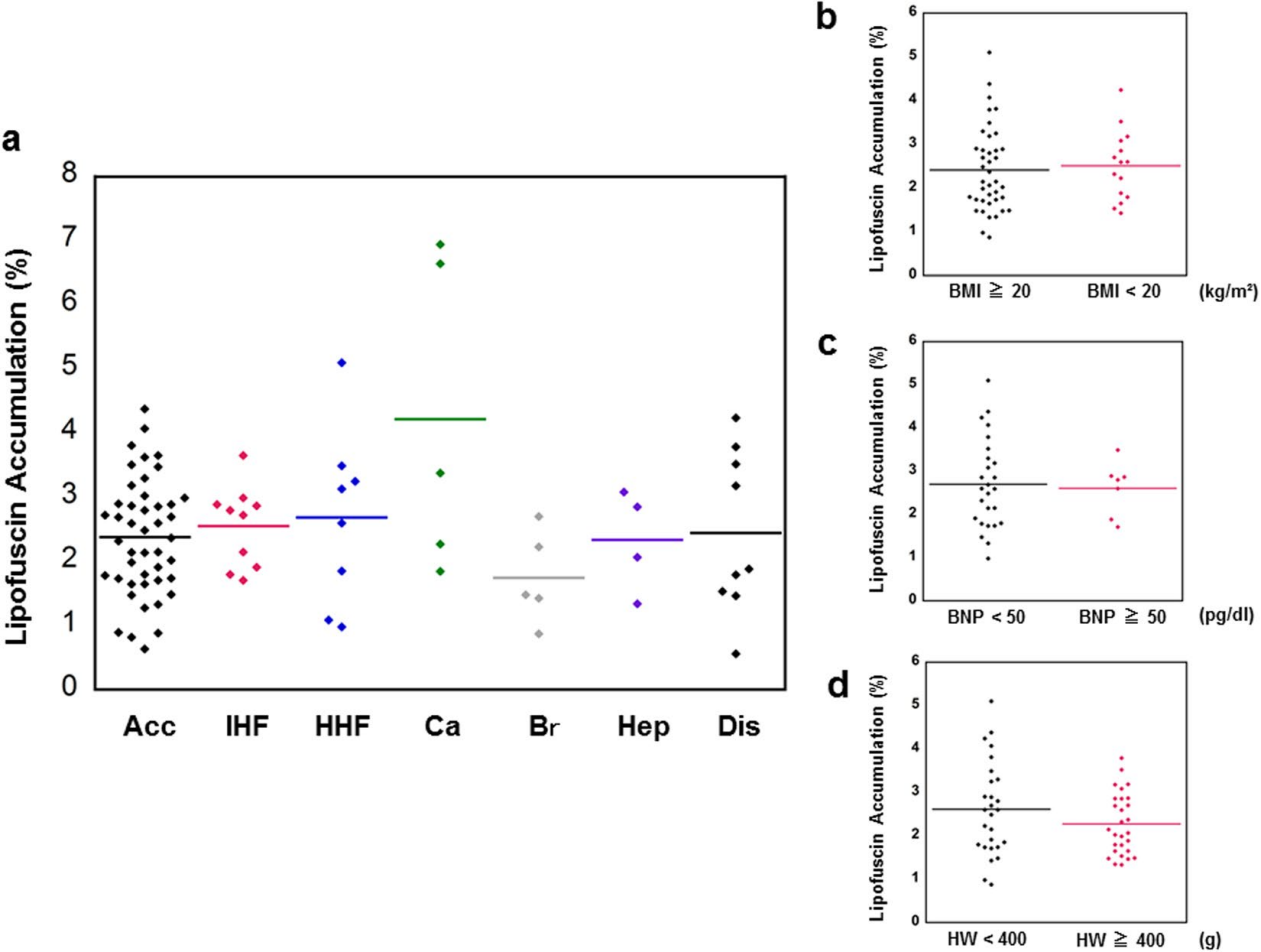
Cardiac lipofuscin accumulation and clinical characteristics. (**a**) Myocardial lipofuscin levels and causes of death. Accidents (Acc; n = 35), ischemic heart failure (IHF; n = 10), hypertrophic heart failure (HHF; n = 8), cancer (Ca; n = 5), brain haemorrhage (Br; n = 5), hepatic failure (Hep; n = 4), and other diseases (Dis; n = 9). (**b**,**c**) Comparison of myocardial lipofuscin accumulation levels in age-matched groups, between high body mass index (BMI; n = 40) and low BMI (n = 15), low brain natriuretic peptide (BNP; n = 26) and high BNP (n = 7), and normal heart weight (n = 28) and cardiac hypertrophy (n = 27). Horizontal bars indicate the mean values.

The mean lipofuscin accumulation rate was 2.3%, 2.6%, 2.7%, 4.2%, 1.7%, 2.3%, and 2.4% in the ACC, IHF, HHF, Ca, Br, Hep, and Dis cases, respectively (Fig. 4a). Two gastric cancer cases showed prominent lipofuscin accumulation, but there was no statistical significance in this sample size. Nutritional status based on BMI, serum BNP level as a biomarker for heart failure, and cardiac hypertrophy did not affect myocardial lipofuscin accumulation (Fig. 4b–d).

### Immunoblotting

**Figure 5:**
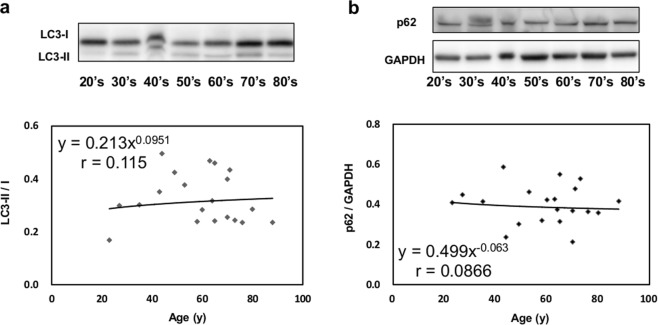
Immunoblotting of cardiac proteins related to autophagy. Normal human hearts from non-cardiac death cases were analysed (n = 20). LC3 and p62 levels were normalized to GAPDH loading control. Representative immunoblots are shown with the larger uncropped immunoblots available as Supplementary Fig S1. No significant changes in myocardial LC3-II/I and p62 levels were associated with ageing.

Twenty non-cardiac death cases (23–88 years) with available frozen tissue were analysed. No significant cardiac age-related changes were observed in the level of autophagosomes as determined by LC3-II/I staining (Fig. 5a) or autophagy clearance as determined by p62 (Fig. 5b, Supplemental Fig. S1).

## Discussion

Ageing is a consecutive process encompassing maturity at youth and degeneration at senium. Ageing speed is affected by genetic background and epigenetic factors including lifestyle and pathological conditions^36,37^. The cellular turnover rate explains the various features of biological ageing. Among the whole organs, cardiac tissue is expected to age in positive correlation with chronological age, because cardiomyocytes are long-lived cells with limited ability to proliferate after birth^38^. Here, we demonstrated for the first time the correlation formula between myocardial lipofuscin accumulation and human chronological age. The resulting correlation was higher than other ageing signs such as changes in pulp/tooth volume^30^ and had almost the same accuracy with shortening of telomere length^28^. Another approach using the combination of DNA methylation patterns could be more accurate in estimating age than myocardial lipofuscin assay^29^, but genome-wide methylation analyses using next-generation sequencing are more expensive. Such DNA analyses are quite useful in the forensics of missing cases where the remains are massively damaged and only a fragment of tissue or blood is available. However, age prediction based on lipofuscin accumulation is applicable as long as the cardiac tissue is observable. Therefore, this technique is practically useful during routine postmortem examinations.

In this study, no significant changes in the lipofuscin amounts were detected in subjects with SCD. The unchanged lipofuscin levels in IHF suggest that the accumulation of lipofuscin has little effect on the cardiac tolerance to ischemia caused by acute coronary occlusion. The HHF cases may also have died of lethal arrhythmia, which is not directly associated with a myocardial lipofuscin deposit. Moreover, an increased BMI, elevated BNP, or cardiac hypertrophy, which are risk factors for SCD, were not found to correlate with the myocardial lipofuscin level. These data suggest that the level of myocardial lipofuscin reflects cardiac chronological ageing more directly than arrhythmogenesis or cardiac dysfunction. However, 40% of Ca patients in this study exhibited massive lipofuscin accumulation. Although the sample size precluded an analysis of statistical significance, some types of metabolic disorders provoked by malignant diseases appear to contribute to lipofuscin accumulation.

Lipofuscin accumulates mainly in the lysosomes. Accordingly, a dysfunction of the autophagy-lysosome system is thought to play a major role in lipofuscinogenesis^4,6^. Besides, many studies have revealed decreased autophagic activity in animal and cell models of ageing^39,40^, which appears to correspond to lipofuscin accumulation. However, chronological age-related autophagic deficiencies in the human heart have not been fully elucidated, and one study reported that autophagic function is maintained even in older patients^41^. Our study also revealed the unchanged amount of autophagosomes and the stable clearance activity of lysosomes during human cardiac ageing. These results support another theory, namely that the autophagosome-lysosome system is not required for lipofuscin formation^10^. Furthermore, impaired mitochondrial fission, the initial step of mitochondria-selective autophagy, contributes to lipofuscinogenesis^7^. Further studies will elucidate the process of lipofuscin formation during ageing, which may involve the simultaneous interactions of several molecular pathways.

We note that our attempts to establish a practical age estimation method at autopsy had some limitations. First, our system of lipofuscin observation with a single filter set may detect false-positive autofluorescence from other cellular components. An overlay of autofluorescence using different filter sets would improve the lipofuscin quantification. However, this technique is costly in a daily post-mortem examination setting. Second, systematic electron microscopy observation is practically difficult because fresh tissues are not always available during forensic autopsies. Therefore, we could not assess changes in mitochondrial morphology in this study.

In conclusion, our study reveals the first correlation between myocardial lipofuscin accumulation and chronological ageing, which appears to be relatively independent of cardiac pathology.

## Supplementary information

Supplemental Dataset 1

## Data Availability

All data generated or analysed during this study are included in this published article (and its Supplementary Information Files).
