# Supplementary material for: Myocardial lipofuscin accumulation in ageing and sudden cardiac death
Source: Sci Rep. 2019 Mar 1;9:3304. doi: 10.1038/s41598-019-40250-0 (PMC6397159; doi:10.1038/s41598-019-40250-0)
Supplement: Supplementary file 1 — Supplemental Dataset 1 [file 41598_2019_40250_MOESM1_ESM.pdf]

Supplemental Data

Myocardial lipofuscin accumulation in ageing and sudden cardiac death

Yu Kakimoto[a*], Chisa Okada[b], Noboru Kawabe[b], Ayumi Sasaki[b], Hideo Tsukamoto[b], Ryoko Nagao[a], Motoki Osawa[a]

[a] Department of Forensic Medicine, Tokai University School of Medicine, Kanagawa, Japan
[b] Support Center for Medical Research and Education, Tokai University, Kanagawa, Japan

*Corresponding author. Department of Forensic Medicine, Tokai University School of Medicine, 143 Shimokasuya, Isehara, Kanagawa 259-1193, Japan
Phone: +81 463 93 1121, Fax: +81 463 92 0284
E-mail: kakimoto@tokai-u.jp (Y. Kakimoto)

Supplemental Table 1

Individual subject's characteristics.

ID	Cause of Death	Age(y)	Sex	Heart Weight(g)	BMI (kg/m2)	BNP (pg/dL)
Acc1	Accident	25	M	448	31.7	not available
Acc2	Accident	20	M	320	18.5	not available
Acc3	Accident	21	F	196	16.9	not available
Acc4	Accident	23	M	306	17.5	18.2
Acc5	Accident	27	M	369	24.3	42.4
Acc6	Accident	44	M	374	22.3	2.0
Acc7	Accident	43	M	356	24.5	2.7
Acc8	Accident	58	F	345	20.8	not available
Acc9	Accident	59	M	370	22.8	not available
Acc10	Accident	67	F	275	19.2	not available
Acc11	Accident	71	M	550	26.6	2.0
Acc12	Accident	43	M	497	28.4	2.0
Acc13	Accident	35	M	339	25.4	17.5
Acc14	Accident	34	F	335	24.4	not available
Acc15	Accident	52	F	320	26.9	not available
Acc16	Accident	93	M	386	22.7	not available
Acc17	Accident	63	M	332	21.7	2.4
Acc18	Accident	49	M	276	18.2	2.0
Acc19	Accident	39	F	277	22.6	not available
Acc20	Accident	63	M	406	22.7	11.1
Acc21	Accident	51	F	246	18.0	2.0
Acc22	Accident	70	F	481	20.0	17.1
Acc23	Accident	53	M	383	25.3	2.0
Acc24	Accident	61	M	531	27.1	not available
Acc25	Accident	70	M	320	22.4	11.4
Acc26	Accident	62	M	460	23.7	not available
Acc27	Accident	91	M	313	15.6	27.4
Acc28	Accident	76	M	361	20.4	5.4
Acc29	Accident	64	M	413	26.1	25.3
Acc30	Accident	81	F	371	26.5	not available
Acc31	Accident	84	M	352	16.8	not available
Acc32	Accident	88	F	259	18.1	155.0
Acc33	Accident	65	M	446	27.6	not available
Acc34	Accident	70	M	448	22.4	9.0
Acc35	Accident	73	M	545	24.0	3.3
IHF1	Ischemic Heart Failure	45	F	477	20.0	111.0
IHF2	Ischemic Heart Failure	41	M	425	27.9	4.7
IHF3	Ischemic Heart Failure	54	M	560	23.9	2.1
IHF4	Ischemic Heart Failure	48	M	408	23.8	3.2
IHF5	Ischemic Heart Failure	82	M	482	23.0	2.9
IHF6	Ischemic Heart Failure	63	M	434	24.8	66.6
IHF7	Ischemic Heart Failure	70	M	360	22.9	118.0
IHF8	Ischemic Heart Failure	65	F	404	21.1	77.1
IHF9	Ischemic Heart Failure	89	F	290	18.3	329.0
IHF10	Ischemic Heart Failure	85	F	250	15.6	89.0
HHF1	Hypertensive Heart Failure	39	M	480	38.1	12.6
HHF2	Aortic Stenosis	82	M	758	25.6	53.8
HHF3	Hypertensive Heart Failure	46	M	754	36.6	not available
HHF4	Hypertensive Heart Failure	80	M	494	19.3	480.0
HHF5	Aortic Stenosis	97	M	444	15.7	146.0
HHF6	Hypertensive Heart Failure	42	M	1104	49.8	not available
HHF7	Hypertensive Heart Failure	74	M	407	26.0	56.6
HHF8	Hypertensive Heart Failure	66	M	596	34.5	2.0
Ca1	Gastric Cancer	67	M	456	21.3	not available
Ca2	Gastric Cancer	61	M	282	19.5	not available
Ca3	Gastric Cancer	65	F	289	11.0	10.5
Ca4	Gastric Cancer	78	M	301	13.6	not available
Ca5	Breast Cancer	51	F	261	16.2	126.0
Br1	Putamen Haemorrhage	65	M	420	20.5	not available
Br2	Pontine Haemorrhage	54	M	458	19.9	not available
Br3	Pontine Haemorrhage	50	M	413	25.1	not available
Br4	Putamen Haemorrhage	66	F	436	15.3	not available
Br5	Thalamic Haemorrhage	60	M	344	17.4	not available
Hep1	Alcohol Cirrhosis	40	M	360	23.4	not available
Hep2	Alcohol Cirrhosis	52	M	395	24.8	not available
Hep3	Alcohol Cirrhosis	49	M	270	18.9	3.1
Hep4	Alcohol Cirrhosis	65	M	302	16.1	16.4
Dis1	Enterogastritis	23	M	224	15.9	not available
Dis2	Sepsis	45	M	381	27.8	not available
Dis3	Ileus	38	M	260	18.5	not available
Dis4	Sepsis	63	M	303	14.1	not available
Dis5	Ileus	72	F	371	15.5	120.0
Dis6	Sepsis	54	F	171	11.5	not available
Dis7	Pneumonia	65	F	218	14.7	10.0
Dis8	Ileus	76	F	312	20.3	41.0
Dis9	Pneumonia	80	M	480	19.4	3.6

Supplemental Table 2

ID	Myocardial Area (miro m^2)	Lipofuscin Area (miro m^2)	Ratio (L/M)
Acc1-1	192,150	1,328	0.0069
Acc1-2	198,665	1,186	0.0060
Acc1-3	188,032	1,056	0.0056
Acc1-4	188,512	1,427	0.0076
Acc1-5	192,349	1,203	0.0063
Acc1-6	205,340	1,307	0.0064
Acc1-7	183,267	1,253	0.0068
Acc1-8	220,819	1,306	0.0059
Acc1-9	196,538	1,382	0.0070
Acc1-10	198,275	1,185	0.0060
Acc2-1	153,599	1,206	0.0079
Acc2-2	126,097	813	0.0064
Acc2-3	109,776	1,217	0.0111
Acc2-4	141,452	935	0.0066
Acc2-5	145,895	1,155	0.0079
Acc2-6	129,162	1,114	0.0086
Acc2-7	150,849	1,527	0.0101
Acc2-8	134,209	1,233	0.0092
Acc2-9	142,305	1,133	0.0080
Acc2-10	131,058	830	0.0063
Acc3-1	168,610	1,514	0.0090
Acc3-2	175,878	1,381	0.0079
Acc3-3	162,020	1,147	0.0071
Acc3-4	163,362	1,119	0.0069
Acc3-5	172,629	1,260	0.0073
Acc3-6	161,370	1,874	0.0116
Acc3-7	158,727	1,631	0.0103
Acc3-8	159,079	1,519	0.0096
Acc3-9	184,865	2,189	0.0118
Acc3-10	152,808	1,084	0.0071
Acc4-1	189,291	1,318	0.0070
Acc4-2	175,699	1,293	0.0074
Acc4-3	182,143	1,776	0.0098
Acc4-4	203,463	1,256	0.0062
Acc4-5	171,658	1,022	0.0060
Acc4-6	192,444	2,613	0.0136
Acc4-7	171,720	1,570	0.0091
Acc4-8	173,221	1,856	0.0107
Acc4-9	183,191	2,282	0.0125
Acc4-10	174,262	1,266	0.0073
Acc5-1	164,942	2,618	0.0159
Acc5-2	203,714	2,474	0.0121
Acc5-3	186,333	2,593	0.0139
Acc5-4	196,010	2,088	0.0107
Acc5-5	192,635	1,850	0.0096
Acc5-6	205,672	3,026	0.0147
Acc5-7	182,120	1,972	0.0108
Acc5-8	205,576	3,019	0.0147
Acc5-9	192,401	2,132	0.0111
Acc5-10	186,965	2,645	0.0141
Acc6-1	150,690	1,987	0.0132
Acc6-2	156,006	2,038	0.0131
Acc6-3	140,034	1,918	0.0137
Acc6-4	156,493	1,715	0.0110
Acc6-5	153,284	2,529	0.0165
Acc6-6	148,402	1,735	0.0117

Acc6-7	146,349	1,879	0.0128
Acc6-8	149,638	2,156	0.0144
Acc6-9	146,812	1,277	0.0087
Acc6-10	139,230	2,527	0.0182
Acc7-1	176,944	2,197	0.0124
Acc7-2	198,479	3,064	0.0154
Acc7-3	200,263	3,901	0.0195
Acc7-4	192,151	2,560	0.0133
Acc7-5	184,082	3,434	0.0187
Acc7-6	207,799	3,258	0.0157
Acc7-7	192,203	2,774	0.0144
Acc7-8	186,364	2,344	0.0126
Acc7-9	192,629	2,430	0.0126
Acc7-10	207,406	2,702	0.0130
Acc8-1	189,510	3,395	0.0179
Acc8-2	184,729	2,640	0.0143
Acc8-3	195,511	3,209	0.0164
Acc8-4	175,520	3,085	0.0176
Acc8-5	201,969	3,139	0.0155
Acc8-6	189,859	2,585	0.0136
Acc8-7	198,866	2,006	0.0101
Acc8-8	204,111	3,230	0.0158
Acc8-9	192,832	2,740	0.0142
Acc8-10	208,098	2,760	0.0133
Acc9-1	191,903	3,005	0.0157
Acc9-2	188,609	2,503	0.0133
Acc9-3	165,473	3,340	0.0202
Acc9-4	186,335	3,088	0.0166
Acc9-5	203,542	3,661	0.0180
Acc9-6	179,537	2,631	0.0147
Acc9-7	174,844	2,530	0.0145
Acc9-8	190,613	3,547	0.0186
Acc9-9	179,114	3,385	0.0189
Acc9-10	177,544	2,536	0.0143
Acc10-1	194,693	3,937	0.0202
Acc10-2	206,505	4,086	0.0198
Acc10-3	205,210	2,687	0.0131
Acc10-4	199,592	2,831	0.0142
Acc10-5	185,396	2,687	0.0145
Acc10-6	198,608	2,845	0.0143
Acc10-7	219,999	4,042	0.0184
Acc10-8	184,831	2,985	0.0162
Acc10-9	195,403	3,682	0.0188
Acc10-10	198,313	3,076	0.0155
Acc11-1	201,931	3,640	0.0180
Acc11-2	194,282	3,138	0.0162
Acc11-3	206,756	3,860	0.0187
Acc11-4	209,554	3,662	0.0175
Acc11-5	217,493	3,645	0.0168
Acc11-6	204,897	3,602	0.0176
Acc11-7	206,418	2,936	0.0142
Acc11-8	200,640	4,139	0.0206
Acc11-9	219,105	4,237	0.0193
Acc11-10	180,191	2,604	0.0145
Acc12-1	155,873	1,796	0.0115
Acc12-2	154,554	1,869	0.0121
Acc12-3	145,295	2,460	0.0169
Acc12-4	149,182	2,829	0.0190
Acc12-5	154,586	2,984	0.0193

Acc12-6	150,239	1,655	0.0110
Acc12-7	142,343	3,008	0.0211
Acc12-8	144,988	2,958	0.0204
Acc12-9	142,677	3,061	0.0215
Acc12-10	139,803	2,930	0.0210
Acc13-1	208,020	3,625	0.0174
Acc13-2	175,537	3,179	0.0181
Acc13-3	199,338	2,869	0.0144
Acc13-4	201,550	2,878	0.0143
Acc13-5	192,734	2,759	0.0143
Acc13-6	190,290	3,937	0.0207
Acc13-7	195,691	4,702	0.0240
Acc13-8	187,534	3,509	0.0187
Acc13-9	201,756	3,586	0.0178
Acc13-10	172,143	3,223	0.0187
Acc14-1	205,781	3,704	0.0180
Acc14-2	213,125	4,261	0.0200
Acc14-3	190,781	3,572	0.0187
Acc14-4	191,918	2,849	0.0148
Acc14-5	223,571	3,890	0.0174
Acc14-6	222,112	5,373	0.0242
Acc14-7	210,972	5,491	0.0260
Acc14-8	194,084	4,171	0.0215
Acc14-9	207,106	4,236	0.0205
Acc14-10	206,502	3,624	0.0176
Acc15-1	147,473	2,903	0.0197
Acc15-2	135,577	3,221	0.0238
Acc15-3	150,051	2,839	0.0189
Acc15-4	156,147	3,326	0.0213
Acc15-5	128,343	3,147	0.0245
Acc15-6	137,981	2,312	0.0168
Acc15-7	148,941	2,430	0.0163
Acc15-8	149,123	2,539	0.0170
Acc15-9	151,874	2,698	0.0178
Acc15-10	156,303	4,063	0.0260
Acc16-1	221,865	4,088	0.0184
Acc16-2	211,877	3,969	0.0187
Acc16-3	193,408	3,060	0.0158
Acc16-4	204,234	3,843	0.0188
Acc16-5	185,584	4,281	0.0231
Acc16-6	211,613	5,359	0.0253
Acc16-7	207,342	5,003	0.0241
Acc16-8	193,134	3,991	0.0207
Acc16-9	210,638	5,061	0.0240
Acc16-10	213,755	5,249	0.0246
Acc17-1	216,483	4,645	0.0215
Acc17-2	199,017	4,024	0.0202
Acc17-3	204,968	4,569	0.0223
Acc17-4	220,441	4,353	0.0197
Acc17-5	213,211	3,861	0.0181
Acc17-6	201,072	4,750	0.0236
Acc17-7	211,716	4,554	0.0215
Acc17-8	218,751	4,437	0.0203
Acc17-9	200,930	4,554	0.0227
Acc17-10	198,744	4,872	0.0245
Acc18-1	223,542	4,543	0.0203
Acc18-2	213,008	4,205	0.0197
Acc18-3	194,988	4,498	0.0231
Acc18-4	190,574	3,775	0.0198

Acc18-5	213,121	4,353	0.0204
Acc18-6	213,637	5,050	0.0236
Acc18-7	197,649	4,565	0.0231
Acc18-8	210,713	5,845	0.0277
Acc18-9	207,258	5,614	0.0271
Acc18-10	198,159	5,378	0.0271
Acc19-1	193,076	3,403	0.0176
Acc19-2	189,180	4,209	0.0222
Acc19-3	196,175	4,321	0.0220
Acc19-4	184,469	3,906	0.0212
Acc19-5	194,887	4,878	0.0250
Acc19-6	205,860	4,568	0.0222
Acc19-7	210,317	4,418	0.0210
Acc19-8	200,084	4,457	0.0223
Acc19-9	194,368	5,570	0.0287
Acc19-10	190,612	6,630	0.0348
Acc20-1	151,259	4,240	0.0280
Acc20-2	144,548	3,926	0.0272
Acc20-3	154,492	3,190	0.0206
Acc20-4	147,398	4,536	0.0308
Acc20-5	154,854	3,424	0.0221
Acc20-6	153,292	2,717	0.0177
Acc20-7	145,587	4,079	0.0280
Acc20-8	149,576	3,865	0.0258
Acc20-9	150,860	3,258	0.0216
Acc20-10	154,258	4,109	0.0266
Acc21-1	136,023	3,887	0.0286
Acc21-2	152,930	4,311	0.0282
Acc21-3	147,819	3,780	0.0256
Acc21-4	138,719	3,722	0.0268
Acc21-5	151,869	3,389	0.0223
Acc21-6	142,149	4,705	0.0331
Acc21-7	153,495	3,346	0.0218
Acc21-8	153,440	2,670	0.0174
Acc21-9	143,267	4,038	0.0282
Acc21-10	143,477	3,927	0.0274
Acc22-1	185,032	3,632	0.0196
Acc22-2	196,169	4,200	0.0214
Acc22-3	200,828	5,730	0.0285
Acc22-4	183,280	4,942	0.0270
Acc22-5	202,228	4,429	0.0219
Acc22-6	202,267	5,680	0.0281
Acc22-7	212,530	5,932	0.0279
Acc22-8	215,235	5,228	0.0243
Acc22-9	215,159	6,230	0.0290
Acc22-10	194,851	6,290	0.0323
Acc23-1	170,138	4,660	0.0274
Acc23-2	145,765	3,543	0.0243
Acc23-3	161,084	4,114	0.0255
Acc23-4	132,790	3,738	0.0281
Acc23-5	161,945	4,858	0.0300
Acc23-6	158,504	4,427	0.0279
Acc23-7	174,130	5,896	0.0339
Acc23-8	131,357	2,864	0.0218
Acc23-9	174,121	4,559	0.0262
Acc23-10	127,523	3,001	0.0235
Acc24-1	152,303	3,122	0.0205
Acc24-2	130,921	3,674	0.0281
Acc24-3	136,852	3,373	0.0246

Acc24-4	152,211	4,071	0.0267
Acc24-5	141,339	3,847	0.0272
Acc24-6	153,595	4,774	0.0311
Acc24-7	151,937	3,334	0.0219
Acc24-8	145,595	4,695	0.0323
Acc24-9	154,463	3,141	0.0203
Acc24-10	120,476	4,402	0.0365
Acc25-1	266,383	6,617	0.0248
Acc25-2	240,418	6,902	0.0287
Acc25-3	270,406	7,198	0.0266
Acc25-4	265,429	8,067	0.0304
Acc25-5	267,261	7,224	0.0270
Acc25-6	259,329	7,631	0.0294
Acc25-7	233,094	7,222	0.0310
Acc25-8	241,558	6,863	0.0284
Acc25-9	250,734	6,612	0.0264
Acc25-10	269,739	8,838	0.0328
Acc26-1	136,350	5,378	0.0394
Acc26-2	149,781	4,588	0.0306
Acc26-3	153,736	4,911	0.0319
Acc26-4	148,309	3,847	0.0259
Acc26-5	142,490	4,674	0.0328
Acc26-6	154,763	3,648	0.0236
Acc26-7	141,160	3,913	0.0277
Acc26-8	149,417	3,302	0.0221
Acc26-9	155,369	4,982	0.0321
Acc26-10	146,588	3,502	0.0239
Acc27-1	247,981	6,606	0.0266
Acc27-2	239,813	7,263	0.0303
Acc27-3	210,970	4,991	0.0237
Acc27-4	265,513	6,907	0.0260
Acc27-5	191,863	7,019	0.0366
Acc27-6	228,937	7,723	0.0337
Acc27-7	209,014	5,377	0.0257
Acc27-8	209,521	6,758	0.0323
Acc27-9	254,674	10,198	0.0400
Acc27-10	255,091	6,950	0.0272
Acc28-1	188,368	6,103	0.0324
Acc28-2	253,039	7,730	0.0305
Acc28-3	233,976	6,213	0.0266
Acc28-4	260,756	9,121	0.0350
Acc28-5	255,555	9,428	0.0369
Acc28-6	260,453	8,415	0.0323
Acc28-7	247,580	8,138	0.0329
Acc28-8	220,080	6,863	0.0312
Acc28-9	262,418	6,837	0.0261
Acc28-10	219,272	7,639	0.0348
Acc29-1	146,282	4,328	0.0296
Acc29-2	151,067	4,229	0.0280
Acc29-3	125,145	6,229	0.0498
Acc29-4	137,991	4,434	0.0321
Acc29-5	144,691	4,559	0.0315
Acc29-6	125,017	5,173	0.0414
Acc29-7	145,368	3,847	0.0265
Acc29-8	123,846	5,007	0.0404
Acc29-9	153,817	4,161	0.0271
Acc29-10	150,385	3,529	0.0235
Acc30-1	175,462	5,879	0.0335
Acc30-2	154,874	5,431	0.0351

Acc30-3	177,269	6,594	0.0372
Acc30-4	179,065	6,141	0.0343
Acc30-5	165,693	5,317	0.0321
Acc30-6	174,075	6,082	0.0349
Acc30-7	161,178	5,116	0.0317
Acc30-8	162,198	5,656	0.0349
Acc30-9	181,992	6,431	0.0353
Acc30-10	163,206	6,305	0.0386
Acc31-1	151,235	3,760	0.0249
Acc31-2	156,132	4,666	0.0299
Acc31-3	134,162	4,870	0.0363
Acc31-4	143,606	5,659	0.0394
Acc31-5	127,393	6,108	0.0479
Acc31-6	136,832	5,039	0.0368
Acc31-7	135,233	4,106	0.0304
Acc31-8	150,712	5,646	0.0375
Acc31-9	151,744	5,352	0.0353
Acc31-10	148,770	4,775	0.0321
Acc32-1	233,028	8,937	0.0384
Acc32-2	222,486	7,865	0.0354
Acc32-3	212,530	8,404	0.0395
Acc32-4	218,059	6,794	0.0312
Acc32-5	218,746	7,855	0.0359
Acc32-6	211,420	7,824	0.0370
Acc32-7	189,847	8,329	0.0439
Acc32-8	208,242	6,303	0.0303
Acc32-9	223,584	8,304	0.0371
Acc32-10	222,268	7,517	0.0338
Acc33-1	153,722	6,684	0.0435
Acc33-2	152,630	6,389	0.0419
Acc33-3	146,959	5,379	0.0366
Acc33-4	148,334	6,205	0.0418
Acc33-5	144,316	6,346	0.0440
Acc33-6	152,618	7,087	0.0464
Acc33-7	153,427	4,629	0.0302
Acc33-8	141,955	4,475	0.0315
Acc33-9	154,892	4,060	0.0262
Acc33-10	141,027	5,500	0.0390
Acc34-1	210,644	9,025	0.0428
Acc34-2	171,503	5,975	0.0348
Acc34-3	176,607	7,466	0.0423
Acc34-4	218,509	10,326	0.0473
Acc34-5	163,624	4,971	0.0304
Acc34-6	171,634	6,544	0.0381
Acc34-7	200,923	8,039	0.0400
Acc34-8	186,663	7,412	0.0397
Acc34-9	159,358	7,450	0.0468
Acc34-10	150,938	6,766	0.0448
Acc35-1	146,896	4,747	0.0323
Acc35-2	168,936	6,722	0.0398
Acc35-3	191,104	7,626	0.0399
Acc35-4	170,220	7,273	0.0427
Acc35-5	160,308	8,176	0.0510
Acc35-6	185,988	7,059	0.0380
Acc35-7	150,636	7,918	0.0526
Acc35-8	194,186	7,881	0.0406
Acc35-9	155,896	7,114	0.0456
Acc35-10	146,293	8,070	0.0552
IHF1-1	145,496	2,540	0.0175

IHF1-2	138,581	2,771	0.0200
IHF1-3	138,430	2,839	0.0205
IHF1-4	151,746	3,278	0.0216
IHF1-5	130,678	2,354	0.0180
IHF1-6	139,489	3,027	0.0217
IHF1-7	154,615	2,561	0.0166
IHF1-8	155,916	1,889	0.0121
IHF1-9	146,852	1,418	0.0097
IHF1-10	144,117	1,921	0.0133
IHF2-1	143,685	1,981	0.0138
IHF2-2	148,058	2,698	0.0182
IHF2-3	139,716	2,940	0.0210
IHF2-4	132,269	3,245	0.0245
IHF2-5	150,313	2,385	0.0159
IHF2-6	144,664	1,585	0.0110
IHF2-7	134,044	2,932	0.0219
IHF2-8	148,107	2,475	0.0167
IHF2-9	147,425	2,662	0.0181
IHF2-10	151,630	2,806	0.0185
IHF3-1	128,792	2,610	0.0203
IHF3-2	125,412	2,128	0.0170
IHF3-3	148,986	2,603	0.0175
IHF3-4	126,605	2,894	0.0229
IHF3-5	156,270	2,532	0.0162
IHF3-6	132,518	2,158	0.0163
IHF3-7	113,461	2,538	0.0224
IHF3-8	139,221	2,031	0.0146
IHF3-9	143,786	3,074	0.0214
IHF3-10	137,740	3,170	0.0230
IHF4-1	169,101	5,418	0.0320
IHF4-2	176,199	3,236	0.0184
IHF4-3	185,286	3,732	0.0201
IHF4-4	168,181	3,181	0.0189
IHF4-5	170,258	3,428	0.0201
IHF4-6	175,516	3,530	0.0201
IHF4-7	172,116	4,668	0.0271
IHF4-8	181,956	3,685	0.0203
IHF4-9	165,322	3,034	0.0184
IHF4-10	175,862	3,418	0.0194
IHF5-1	156,155	3,707	0.0237
IHF5-2	133,225	4,643	0.0348
IHF5-3	142,004	4,116	0.0290
IHF5-4	143,568	3,955	0.0275
IHF5-5	138,990	3,755	0.0270
IHF5-6	152,638	4,250	0.0278
IHF5-7	144,556	2,919	0.0202
IHF5-8	144,924	3,608	0.0249
IHF5-9	137,909	3,190	0.0231
IHF5-10	131,590	4,451	0.0338
IHF6-1	170,408	4,048	0.0238
IHF6-2	178,662	4,959	0.0278
IHF6-3	184,432	5,380	0.0292
IHF6-4	187,202	5,895	0.0315
IHF6-5	183,079	5,928	0.0324
IHF6-6	196,276	5,725	0.0292
IHF6-7	205,128	5,055	0.0246
IHF6-8	181,181	5,059	0.0279
IHF6-9	193,221	5,510	0.0285
IHF6-10	199,411	5,034	0.0252

IHF7-1	141,535	6,053	0.0428
IHF7-2	149,333	4,784	0.0320
IHF7-3	145,167	3,978	0.0274
IHF7-4	148,495	4,707	0.0317
IHF7-5	153,907	3,295	0.0214
IHF7-6	147,325	3,568	0.0242
IHF7-7	154,160	3,908	0.0254
IHF7-8	141,967	3,649	0.0257
IHF7-9	155,985	3,753	0.0241
IHF7-10	149,794	4,793	0.0320
IHF8-1	150,510	3,532	0.0235
IHF8-2	145,935	4,538	0.0311
IHF8-3	137,224	3,625	0.0264
IHF8-4	152,893	3,877	0.0254
IHF8-5	152,185	4,774	0.0314
IHF8-6	137,503	5,046	0.0367
IHF8-7	155,273	4,359	0.0281
IHF8-8	154,948	5,097	0.0329
IHF8-9	131,914	3,418	0.0259
IHF8-10	147,622	4,072	0.0276
IHF9-1	154,366	3,812	0.0247
IHF9-2	150,105	4,625	0.0308
IHF9-3	152,792	3,415	0.0224
IHF9-4	152,831	4,486	0.0294
IHF9-5	145,455	4,086	0.0281
IHF9-6	146,353	4,830	0.0330
IHF9-7	155,256	4,834	0.0311
IHF9-8	155,310	5,169	0.0333
IHF9-9	149,203	5,541	0.0371
IHF9-10	146,037	4,277	0.0293
IHF10-1	208,136	6,435	0.0309
IHF10-2	222,829	9,617	0.0432
IHF10-3	182,691	7,672	0.0420
IHF10-4	185,809	6,790	0.0365
IHF10-5	210,958	7,158	0.0339
IHF10-6	189,399	7,454	0.0394
IHF10-7	180,015	7,947	0.0441
IHF10-8	219,381	9,000	0.0410
IHF10-9	222,239	6,185	0.0278
IHF10-10	208,068	5,353	0.0257
HHF1-1	218,175	1,688	0.0077
HHF1-2	227,459	2,013	0.0089
HHF1-3	231,345	2,635	0.0114
HHF1-4	230,470	3,022	0.0131
HHF1-5	252,835	2,559	0.0101
HHF1-6	238,492	2,109	0.0088
HHF1-7	232,785	1,903	0.0082
HHF1-8	235,548	2,174	0.0092
HHF1-9	254,732	2,706	0.0106
HHF1-10	239,749	2,342	0.0098
HHF2-1	266,906	3,222	0.0121
HHF2-2	258,578	2,342	0.0091
HHF2-3	279,605	2,393	0.0086
HHF2-4	277,826	1,680	0.0060
HHF2-5	259,845	2,248	0.0087
HHF2-6	238,364	3,808	0.0160
HHF2-7	231,826	3,055	0.0132
HHF2-8	223,967	3,333	0.0149
HHF2-9	256,043	2,404	0.0094

HHF2-10	207,265	2,320	0.0112
HHF3-1	232,988	5,576	0.0239
HHF3-2	233,500	6,361	0.0272
HHF3-3	216,340	2,138	0.0099
HHF3-4	200,638	4,149	0.0207
HHF3-5	212,630	3,319	0.0156
HHF3-6	195,984	2,375	0.0121
HHF3-7	178,307	3,123	0.0175
HHF3-8	215,922	4,507	0.0209
HHF3-9	247,659	3,485	0.0141
HHF3-10	193,922	4,442	0.0229
HHF4-1	154,929	3,129	0.0202
HHF4-2	149,435	4,030	0.0270
HHF4-3	146,433	4,079	0.0279
HHF4-4	155,949	3,713	0.0238
HHF4-5	155,121	3,367	0.0217
HHF4-6	143,712	3,848	0.0268
HHF4-7	144,916	3,684	0.0254
HHF4-8	151,887	4,646	0.0306
HHF4-9	146,047	4,223	0.0289
HHF4-10	152,641	4,164	0.0273
HHF5-1	231,821	6,801	0.0293
HHF5-2	222,587	4,975	0.0224
HHF5-3	184,525	4,340	0.0235
HHF5-4	236,111	4,706	0.0199
HHF5-5	222,201	4,053	0.0182
HHF5-6	244,840	8,368	0.0342
HHF5-7	239,867	5,305	0.0221
HHF5-8	220,674	3,389	0.0154
HHF5-9	262,363	28,275	0.1078
HHF5-10	232,942	4,814	0.0207
HHF6-1	202,808	7,306	0.0360
HHF6-2	229,806	7,413	0.0323
HHF6-3	231,705	8,529	0.0368
HHF6-4	211,956	8,622	0.0407
HHF6-5	236,448	5,689	0.0241
HHF6-6	204,625	6,006	0.0293
HHF6-7	223,113	7,110	0.0319
HHF6-8	196,569	5,519	0.0281
HHF6-9	237,651	8,146	0.0343
HHF6-10	231,554	7,224	0.0312
HHF7-1	143,086	4,784	0.0334
HHF7-2	146,995	5,316	0.0362
HHF7-3	151,028	5,841	0.0387
HHF7-4	145,774	5,957	0.0409
HHF7-5	144,133	4,349	0.0302
HHF7-6	140,413	3,781	0.0269
HHF7-7	151,040	3,966	0.0263
HHF7-8	141,696	6,514	0.0460
HHF7-9	147,602	5,070	0.0344
HHF7-10	134,915	4,822	0.0357
HHF8-1	207,620	10,948	0.0527
HHF8-2	211,965	11,124	0.0525
HHF8-3	226,492	13,904	0.0614
HHF8-4	224,124	11,577	0.0517
HHF8-5	224,892	8,375	0.0372
HHF8-6	223,199	10,324	0.0463
HHF8-7	219,599	8,739	0.0398
HHF8-8	211,262	10,985	0.0520

HHF8-9	212,783	15,891	0.0747
HHF8-10	246,001	10,167	0.0413
Ca1-1	133,815	2,285	0.0171
Ca1-2	134,718	2,482	0.0184
Ca1-3	133,344	3,470	0.0260
Ca1-4	153,552	2,376	0.0155
Ca1-5	129,465	2,570	0.0199
Ca1-6	147,572	2,672	0.0181
Ca1-7	138,615	1,972	0.0142
Ca1-8	149,146	2,375	0.0159
Ca1-9	133,392	2,834	0.0212
Ca1-10	138,940	2,542	0.0183
Ca2-1	155,937	3,462	0.0222
Ca2-2	153,704	2,989	0.0194
Ca2-3	150,883	3,133	0.0208
Ca2-4	154,193	3,371	0.0219
Ca2-5	155,967	3,386	0.0217
Ca2-6	144,417	4,195	0.0290
Ca2-7	154,662	3,131	0.0202
Ca2-8	155,583	3,532	0.0227
Ca2-9	155,677	4,491	0.0288
Ca2-10	144,435	2,907	0.0201
Ca3-1	176,597	5,894	0.0334
Ca3-2	187,294	7,231	0.0386
Ca3-3	179,128	6,300	0.0352
Ca3-4	179,451	6,204	0.0346
Ca3-5	174,435	6,290	0.0361
Ca3-6	174,497	5,774	0.0331
Ca3-7	186,519	5,370	0.0288
Ca3-8	185,054	5,604	0.0303
Ca3-9	187,816	6,593	0.0351
Ca3-10	189,727	6,162	0.0325
Ca4-1	146,806	10,329	0.0704
Ca4-2	152,492	11,502	0.0754
Ca4-3	145,947	11,120	0.0762
Ca4-4	152,503	9,711	0.0637
Ca4-5	147,547	7,791	0.0528
Ca4-6	148,951	12,079	0.0811
Ca4-7	154,294	10,009	0.0649
Ca4-8	155,263	10,026	0.0646
Ca4-9	150,781	7,795	0.0517
Ca4-10	149,738	9,487	0.0634
Ca5-1	209,883	13,441	0.0640
Ca5-2	238,875	14,922	0.0625
Ca5-3	245,564	17,950	0.0731
Ca5-4	179,105	11,640	0.0650
Ca5-5	144,529	18,286	0.1265
Ca5-6	206,070	12,704	0.0616
Ca5-7	227,037	13,985	0.0616
Ca5-8	249,435	14,741	0.0591
Ca5-9	238,861	17,406	0.0729
Ca5-10	272,267	12,975	0.0477
Br1-1	245,376	1,903	0.0078
Br1-2	260,811	1,844	0.0071
Br1-3	244,893	1,489	0.0061
Br1-4	241,624	2,139	0.0089
Br1-5	237,314	2,262	0.0095
Br1-6	258,302	2,957	0.0114
Br1-7	261,720	2,555	0.0098

Br1-8	256,938	2,157	0.0084
Br1-9	248,607	2,151	0.0087
Br1-10	258,777	2,580	0.0100
Br2-1	234,497	2,976	0.0127
Br2-2	231,731	3,230	0.0139
Br2-3	242,086	3,144	0.0130
Br2-4	248,181	5,021	0.0202
Br2-5	216,033	2,392	0.0111
Br2-6	239,384	3,671	0.0153
Br2-7	242,213	3,752	0.0155
Br2-8	220,671	3,413	0.0155
Br2-9	224,941	2,803	0.0125
Br2-10	235,250	3,079	0.0131
Br3-1	219,853	2,852	0.0130
Br3-2	251,944	2,096	0.0083
Br3-3	234,167	3,735	0.0160
Br3-4	229,586	3,915	0.0171
Br3-5	226,041	2,456	0.0109
Br3-6	220,589	4,375	0.0198
Br3-7	211,238	3,391	0.0161
Br3-8	250,308	3,597	0.0144
Br3-9	253,874	4,012	0.0158
Br3-10	252,157	4,256	0.0169
Br4-1	259,937	7,012	0.0270
Br4-2	254,688	3,750	0.0147
Br4-3	266,710	5,385	0.0202
Br4-4	251,275	4,374	0.0174
Br4-5	228,650	6,860	0.0300
Br4-6	243,019	4,718	0.0194
Br4-7	251,724	6,591	0.0262
Br4-8	249,602	5,327	0.0213
Br4-9	268,553	7,204	0.0268
Br4-10	266,156	5,220	0.0196
Br5-1	226,611	5,990	0.0264
Br5-2	217,513	7,512	0.0345
Br5-3	218,973	8,076	0.0369
Br5-4	235,334	6,340	0.0269
Br5-5	266,997	5,387	0.0202
Br5-6	254,384	7,164	0.0282
Br5-7	267,154	7,207	0.0270
Br5-8	263,854	5,743	0.0218
Br5-9	269,057	7,037	0.0262
Br5-10	251,316	5,555	0.0221
Hep1-1	207,796	2,526	0.0122
Hep1-2	195,539	2,633	0.0135
Hep1-3	187,448	2,051	0.0109
Hep1-4	199,153	3,652	0.0183
Hep1-5	192,703	1,514	0.0079
Hep1-6	204,015	1,632	0.0080
Hep1-7	201,680	4,056	0.0201
Hep1-8	202,061	2,885	0.0143
Hep1-9	203,123	2,763	0.0136
Hep1-10	186,889	2,924	0.0156
Hep2-1	149,372	3,004	0.0201
Hep2-2	149,542	3,249	0.0217
Hep2-3	155,521	3,215	0.0207
Hep2-4	153,924	3,307	0.0215
Hep2-5	151,083	3,120	0.0206
Hep2-6	150,485	2,776	0.0184

Hep2-7	155,651	2,743	0.0176
Hep2-8	153,160	2,988	0.0195
Hep2-9	144,442	3,580	0.0248
Hep2-10	153,400	3,292	0.0215
Hep3-1	150,449	4,294	0.0285
Hep3-2	177,444	4,676	0.0263
Hep3-3	148,175	4,756	0.0321
Hep3-4	154,813	5,269	0.0340
Hep3-5	160,733	4,209	0.0262
Hep3-6	165,603	4,673	0.0282
Hep3-7	166,833	5,052	0.0303
Hep3-8	175,068	4,427	0.0253
Hep3-9	154,169	4,144	0.0269
Hep3-10	153,486	4,186	0.0273
Hep4-1	182,754	6,432	0.0352
Hep4-2	179,365	8,319	0.0464
Hep4-3	186,370	6,408	0.0344
Hep4-4	213,822	6,772	0.0317
Hep4-5	218,237	6,368	0.0292
Hep4-6	199,555	5,070	0.0254
Hep4-7	198,400	5,259	0.0265
Hep4-8	210,512	4,953	0.0235
Hep4-9	214,483	6,076	0.0283
Hep4-10	210,967	5,853	0.0277
Dis1-1	214,989	1,500	0.0070
Dis1-2	196,330	1,316	0.0067
Dis1-3	237,583	2,152	0.0091
Dis1-4	248,584	1,371	0.0055
Dis1-5	241,758	1,079	0.0045
Dis1-6	237,117	1,184	0.0050
Dis1-7	243,110	1,705	0.0070
Dis1-8	229,044	456	0.0020
Dis1-9	225,996	770	0.0034
Dis1-10	238,222	1,520	0.0064
Dis2-1	238,640	3,483	0.0146
Dis2-2	246,609	3,987	0.0162
Dis2-3	250,464	3,614	0.0144
Dis2-4	226,303	3,547	0.0157
Dis2-5	224,303	3,601	0.0161
Dis2-6	233,240	3,136	0.0134
Dis2-7	250,368	3,004	0.0120
Dis2-8	252,488	2,991	0.0118
Dis2-9	236,418	4,344	0.0184
Dis2-10	223,447	3,107	0.0139
Dis3-1	257,233	2,985	0.0116
Dis3-2	252,310	3,645	0.0144
Dis3-3	239,438	3,445	0.0144
Dis3-4	230,341	2,712	0.0118
Dis3-5	246,482	3,719	0.0151
Dis3-6	236,072	4,424	0.0187
Dis3-7	250,046	4,126	0.0165
Dis3-8	239,648	3,351	0.0140
Dis3-9	236,915	4,033	0.0170
Dis3-10	265,427	5,245	0.0198
Dis4-1	218,720	4,384	0.0200
Dis4-2	246,520	3,788	0.0154
Dis4-3	215,837	3,415	0.0158
Dis4-4	238,038	4,255	0.0179
Dis4-5	234,689	4,667	0.0199

Dis4-6	254,613	3,527	0.0139
Dis4-7	251,656	4,815	0.0191
Dis4-8	225,546	4,447	0.0197
Dis4-9	217,578	4,215	0.0194
Dis4-10	256,734	4,739	0.0185
Dis5-1	158,378	3,406	0.0215
Dis5-2	221,414	4,184	0.0189
Dis5-3	223,121	4,307	0.0193
Dis5-4	220,279	3,876	0.0176
Dis5-5	169,379	3,382	0.0200
Dis5-6	203,818	3,414	0.0168
Dis5-7	189,160	3,493	0.0185
Dis5-8	200,621	4,495	0.0224
Dis5-9	190,812	3,009	0.0158
Dis5-10	209,823	3,707	0.0177
Dis6-1	234,946	8,061	0.0343
Dis6-2	212,530	7,683	0.0361
Dis6-3	222,299	7,870	0.0354
Dis6-4	215,864	4,969	0.0230
Dis6-5	218,732	7,460	0.0341
Dis6-6	216,038	7,752	0.0359
Dis6-7	214,185	7,277	0.0340
Dis6-8	217,062	6,409	0.0295
Dis6-9	222,284	6,933	0.0312
Dis6-10	214,980	5,204	0.0242
Dis7-1	215,845	9,509	0.0441
Dis7-2	223,252	9,373	0.0420
Dis7-3	209,432	7,100	0.0339
Dis7-4	210,295	8,485	0.0403
Dis7-5	201,749	6,633	0.0329
Dis7-6	207,481	7,576	0.0365
Dis7-7	199,688	6,540	0.0328
Dis7-8	216,616	5,669	0.0262
Dis7-9	202,389	5,750	0.0284
Dis7-10	200,856	7,013	0.0349
Dis8-1	146,602	4,624	0.0315
Dis8-2	151,393	4,582	0.0303
Dis8-3	148,622	5,550	0.0373
Dis8-4	154,625	4,026	0.0260
Dis8-5	154,136	6,307	0.0409
Dis8-6	147,429	6,578	0.0446
Dis8-7	154,653	5,197	0.0336
Dis8-8	151,406	6,918	0.0457
Dis8-9	149,896	6,315	0.0421
Dis8-10	149,477	7,068	0.0473
Dis9-1	176,021	6,743	0.0383
Dis9-2	196,129	7,457	0.0380
Dis9-3	190,893	7,993	0.0419
Dis9-4	194,046	8,151	0.0420
Dis9-5	182,163	7,030	0.0386
Dis9-6	190,927	8,306	0.0435
Dis9-7	178,755	7,053	0.0395
Dis9-8	178,482	9,284	0.0520
Dis9-9	188,266	8,613	0.0458
Dis9-10	179,908	8,011	0.0445

Supplemental Figure S1.

Immunoblotting of cardiac LC3 and p62. GAPDH was used as roading control. Exposure time was set at 1min for LC3 and p62 detection, and 10sec for GAPDH detection. Dashed lines indicate cropped immunoblots presented in Fig5.

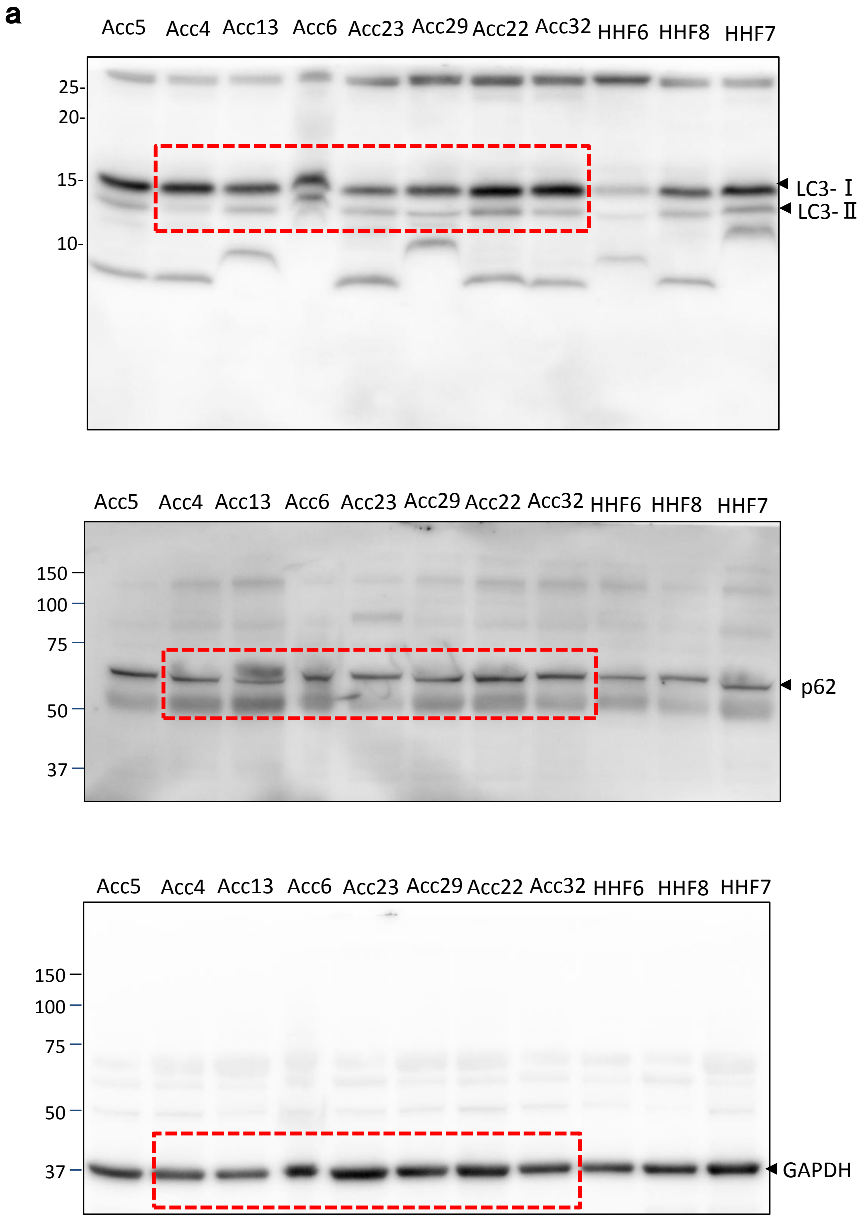

b

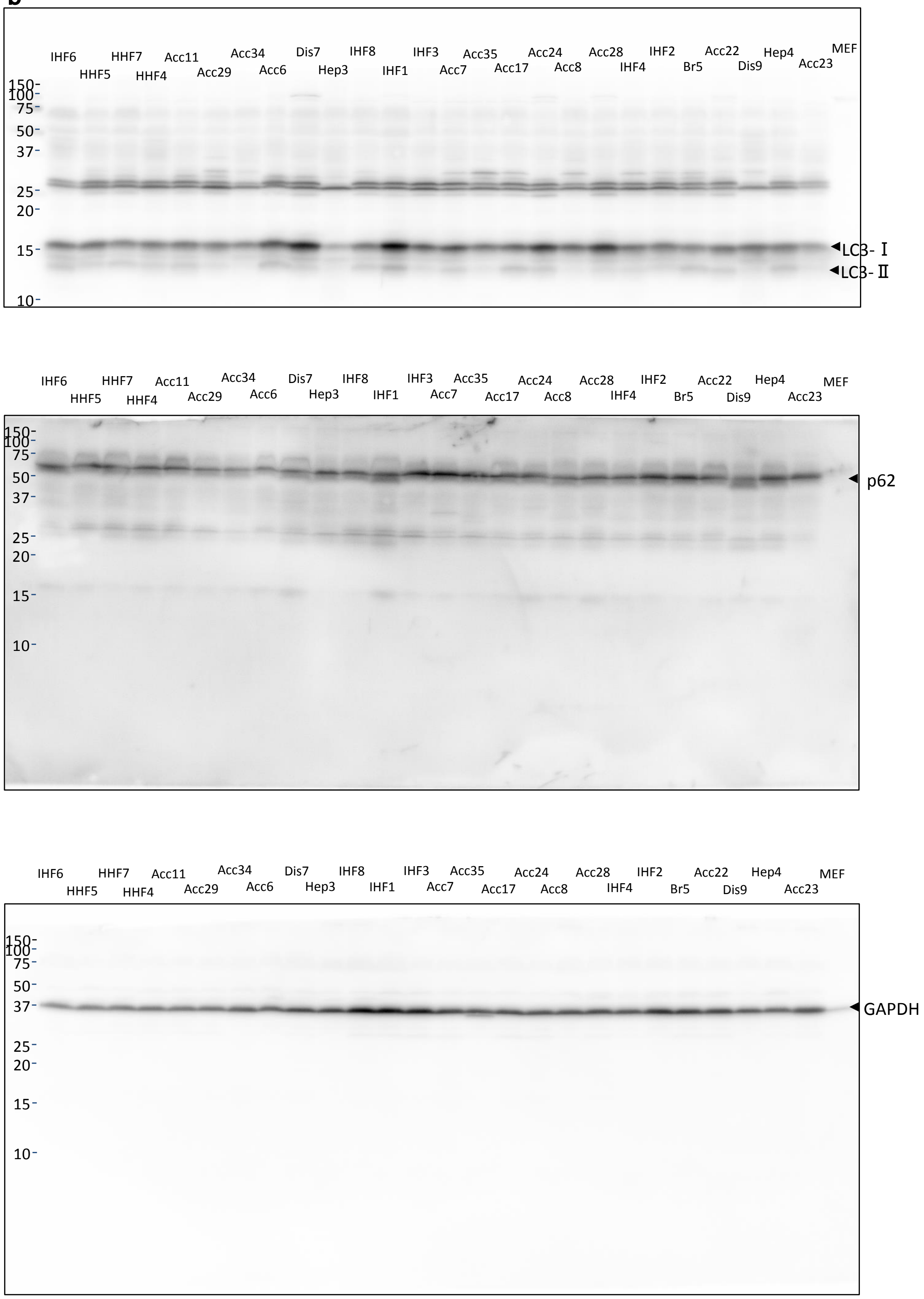
IHF6 HHF5 HHF7 HHF4 Acc11 Acc29 Acc34 Acc6 Dis7 Hep3 IHF8 IHF1 IHF3 Acc7 Acc35 Acc17 Acc24 Acc8 Acc28 IHF4 IHF2 Br5 Acc22 Dis9 Hep4 Acc23 MEF
150-
100-
75-
50-
37-
25-
20-
15-
10-
LC3- Ⅰ
LC3- Ⅱ
IHF6 HHF5 HHF7 HHF4 Acc11 Acc29 Acc34 Acc6 Dis7 Hep3 IHF8 IHF1 IHF3 Acc7 Acc35 Acc17 Acc24 Acc8 Acc28 IHF4 IHF2 Br5 Acc22 Dis9 Hep4 Acc23 MEF
150-
100-
75-
50-
37-
25-
20-
15-
10-
p62
IHF6 HHF5 HHF7 HHF4 Acc11 Acc29 Acc34 Acc6 Dis7 Hep3 IHF8 IHF1 IHF3 Acc7 Acc35 Acc17 Acc24 Acc8 Acc28 IHF4 IHF2 Br5 Acc22 Dis9 Hep4 Acc23 MEF
150-
100-
75-
50-
37-
25-
20-
15-
10-
GAPDH
